# E-Cigarette Use, Systemic Inflammation, and Depression

**DOI:** 10.3390/ijerph181910402

**Published:** 2021-10-02

**Authors:** Kayla Rae Farrell, Emma Karey, Shu Xu, Grace Gibbon, Terry Gordon, Michael Weitzman

**Affiliations:** 1 Department of Environmental Medicine, New York University Grossman School of Medicine, New York, NY 10010, USA; kayla.farrell@nyulangone.org (K.R.F.); emma.karey@nyulangone.org (E.K.); terry.gordon@nyulangone.org (T.G.); 2 Department of Biostatistics, School of Global Public Health, New York University, New York, NY 10003, USA; sx5@nyu.edu; 3 Department of Epidemiology, School of Global Public Health, New York University, New York, NY 10003, USA; gg2260@nyu.edu; 4 Department of Social and Behavioral Sciences, School of Global Public Health, New York University, New York, NY 10003, USA; 5 Department of Pediatrics, New York University Grossman School of Medicine, New York, NY 10016, USA

**Keywords:** e-cigarettes, inflammation, depression, C-reactive protein

## Abstract

Background: E-cigarette use (vaping) is an emerging public health problem. Depression has been found to be associated with e-cigarette use, and vaping and depression are each associated with elevated systemic inflammation. To date, the role of inflammation in the relationship between vaping and depression has not been explored. Objective: To assess the independent associations between e-cigarette use, depression, and inflammation, and to investigate whether the likelihood of depression among current e-cigarette users is associated with systemic inflammation. Methods: Nationally representative NHANES data from 2015–2018 were used (*n* = 4961). Systemic inflammation was defined as serum C-reactive protein (CRP) ≥ 8.0 mg/L. Depressed individuals were characterized by a score ≥ 10 on the Patient Health Questionnaire-9 (PHQ-9). Current e-cigarette users were defined as individuals who vaped at least once in the past 30 days and these individuals were stratified by use: exclusive users (reported smoking less than 100 combustible cigarettes in their lifetime), dual users (reported current use of electronic and combustible cigarettes), and e-cigarette users who were previous smokers. Bivariate analyses were used to assess independent associations between vaping, depression, and inflammation; and weighted logistic regression analyses adjusting for BMI, sex, and economic status were used to determine the odds ratios (ORs) for depression by e-cigarette category stratified by differential CRP levels. Results: Depression occurred in 16.7% of all e-cigarette users vs. 5.0% of those who never used e-cigarettes (*p* < 0.001). In adjusted analyses, the following elevated ORs were found: all current e-cigarette users with CRP <8 = 3.37 (95% CI: 2.06, 5.51) vs. CRP ≥8 = 6.70 (2.48, 18.11); exclusive e-cigarette users with CRP <8 = 1.91 (0.78, 4.69) vs. those with CRP ≥8 = 5.09 (1.44, 18.02); and dual users with CRP <8 = 4.31 (2.35, 7.89) vs. those with CRP ≥8 = 7.37 (1.85, 29.41). These ORs indicate that depression is associated with each category of e-cigarette use; however, we found this association did not vary by systemic inflammation level (interaction *p*-values > 0.05). Conclusion: While a pattern of greater ORs for depression among e-cigarette users with elevated CRP provides provocative findings that might suggest a potential role of inflammation in the association between vaping and depression, we failed to find evidence that inflammation clearly moderates this association. While it is possible that depression among e-cigarette users may be influenced by systemic inflammation, a reproduction of the current study is necessary among a larger cohort to elucidate the effect of inflammation on depression among e-cigarette users.

## 1. Introduction

While combustible cigarette smoking continues to decrease in the United States, there has been a startling rise in electronic cigarette (e-cigarette) use (called “vaping”) [1]. The public health community is divided between those who consider e-cigarettes a less harmful nicotine delivery device (compared to cigarette smoking), and those who are concerned about the potential unknown risks associated with vaping. Studies have shown adolescent e-cigarette users are more likely to experiment and subsequently use traditional cigarettes compared to adolescents who are non-users [2]. Similar to the ongoing potential for an “e-cigarette epidemic”, depression is recognized as a serious global public health concern that is estimated to affect 322 million people worldwide (4.4% of the global population as of 2015) [3]. Furthermore, depressive disorders accounted for a global total that exceeded 50 million Years Lived with Disability (YLD) in 2015 [3]. Clearly, depression and vaping are two pernicious public health problems.

There is a well-established link between depression and the use of traditional combustible tobacco products at increased rates and intensity (i.e., depressed individuals are more likely to smoke cigarettes and smoke more cigarettes per day). While e-cigarettes are relatively new to the US market (<15 years), a similar relationship has been observed between depression and e-cigarette use [4,5,6]. While many studies group together all current e-cigarette users, regardless of current or former smoking history, one previous cross-sectional study that used Behavioral Risk Factor Surveillance System data from 2016–2017 found that never cigarette smokers who used e-cigarettes were two times more likely to be depressed compared to the reference group of non-vaping individuals [6]. This suggests that e-cigarettes are associated with depression even in the absence of current or former combustible cigarette smoking, although this clearly warrants further investigation. Despite the strong association between combustible tobacco-use and depression and the emerging evidence supporting an association between e-cigarette use and depression, the mechanisms underlying these associations remain poorly understood.

An increasing body of literature has identified a link between e-cigarette use and inflammation (in the lung and other organs) [7]. The data from both laboratory and population-based studies suggest that vaping may promote inflammation similarly to traditional cigarettes [8,9,10,11,12], as current cigarette smokers have been found to present significantly higher levels of C-reactive protein (CRP) and other inflammatory markers [13]. Systemic inflammation is a known risk factor for myriad health conditions, including depression [14,15,16]. Separately, depressive symptoms are associated with pro-inflammatory processes, suggesting the utility of inflammatory proteins such as CRP as a potential systemic biomarker of major depressive disorders [17,18,19,20].

While previous work has sought out to assess the cumulative impact of inflammatory markers on the mental health of smokers [21,22,23], to our knowledge, no research has examined the potential moderating effect of systemic inflammation on depression likelihood among current e-cigarette users. We set out to accomplish the following: (1) verify independent associations between e-cigarette use and depression, depression and inflammation, e-cigarette use and inflammation; (2) assess whether among all current e-cigarette users, odds of depression varies by stratified CRP level; (3) investigate whether the association between different categories of e-cigarette use (e.g., exclusive e-cigarette users, dual users, etc.) and depression varies by stratified CRP level; and (4) determine whether inflammation moderates the association between e-cigarette use and depression. Self-reported e-cigarette use, depression survey responses, and serologic CRP data from the nationally representative National Health and Nutrition Examination Survey (NHANES) were used to investigate these associations.

## 2. Methods

Data from the 2015–2016 and 2017–2018 NHANES survey cycles were merged (*n* = 11,848 individuals 18 years and older) to evaluate the relationship between depression, e-cigarette use, and systemic inflammation. Data from these two cycles were merged to increase the sample size and enhance the statistical power for detecting significant associations between e-cigarette use, inflammation, and depression. Any participant missing data for C-reactive protein (*n* = 1332), depression (*n* = 1646), poverty status (*n* = 1511), body mass index (*n* = 752), or ever e-cigarette use (*n* = 4) was excluded from analyses (some participants were missing data for two or more of these variables). Participants with a history of e-cigarette use or traditional smoking that did not fit into our defined categories of interest were also excluded (*n* = 4760), leaving a total of 4961 participants (Table 1 shows the characteristics of the final sample).

### 2.1. E-Cigarette Users

E-cigarette users were identified as individuals who answered ‘yes’ to the question: “Have you ever used an e-cigarette?” Within this population, current e-cigarette users (*n* = 443) were identified as individuals who reported using an e-cigarette at least one day in the last 30. Individuals that reported ever using an e-cigarette, but had not used one within the past 30 days were excluded from analysis (*n* = 1614) as limited information made it difficult to discern whether these individuals were once regular e-cigarette users who had made the decision to stop vaping, or were simply individuals who had tried vaping one time/a few times recreationally. Among current e-cigarette users, 24.8% (*n* = 110) were exclusive e-cigarette users (responded ‘no’ when asked about smoking a minimum of 100 cigarettes in their lifetime); 54.4% (*n* = 241) were dual users (current e-cigarette users who also reported smoking a minimum of 100 traditional combustible cigarettes and reported currently smoking “every day” or “some days”); and 20.8% (*n* = 92) were former smokers using e-cigarettes exclusively. Former smokers included those who smoked a minimum of 100 cigarettes in their lifetime, but presently reported “not at all” for current smoking.

### 2.2. Reference Group

The *reference group* (*n* = 4518) included individuals who answered ‘no’ to having ever used an e-cigarette AND responded ‘no’ when asked about having smoked a minimum of 100 cigarettes in their lifetime (non-smoking/never-vaping persons). Individuals with no history of e-cigarette use who had used more than 100 cigarettes in their lifetime (*n* = 3146) were excluded from the reference group (smoking/never-vaping persons). Previously, we contributed to existing smoking, inflammation, and depression literature [21,22,23] when we showed that current and former cigarette smokers with elevated CRP were 3.21 times more likely to be depressed than nonsmokers (based on participants from the 2015–2016 NHANES dataset) [24]. Therefore, we chose to exclude any non-vaping individuals that reported smoking at least 100 cigarettes over the course of their lives from the reference group for these analyses.

### 2.3. Depression

Depression was assessed using the Patient Health Questionnaire-9 (PHQ-9), a 9-question screener that touches upon key aspects of clinical depression as outlined in the Diagnostic and Statistical Manual of Mental Disorders (DSM), and has been validated both in clinical settings and epidemiological surveys [25,26,27]. Each question is scored on a four-point Likert scale from (0 = not at all; 1 = several days; 2 = more than half the days; 3 = nearly every day), and the scores are summed to yield a total PHQ-9 score between 0 and 27. Consistent with other studies, individuals with a cumulative PHQ-9 score ≥ 10 were characterized as depressed [25].

### 2.4. Inflammation

High sensitivity C-reactive protein (CRP) was used as the biomarker for systemic inflammation [28]. Serum samples were obtained from participants as part of the NHANES biospecimen collection protocol. CRP was measured using a Beckman Coulter UniCel DxC 660i Synchron Access chemistry analyzer (DxC 660i; Beckman Coulter, Inc., Fullerton, CA USA) for the 2015–2016 survey cycle and a Roche Cobas 6000 chemistry analyzer (Cobas 6000; Roche Diagnostics, Indianapolis, IN USA) for the 2017–2018 survey cycle following laboratory procedures detailed by NHANES [29,30]. NHANES has reported that 2017–2018 CRP values were 19.6% lower than values measured in 2015–2016 using the DxC 660i instrument. In order to merge the 2015–2016 and 2017–2018 data for this analysis, the following recommended regression equation was applied to values ≤ 23 mg/L (*n* = 2510) measured with the DxC 660i instrument: Updated DxC 660i value = (0.8695 × Original DxC 660i value) + 0.2954 [30]. The 58 values over 23 mg/L remained unchanged as NHANES did not report a recommended adjustment for those values [30]. Serum CRP level was dichotomized into two groups: reference (<8.0 mg/L serum CRP) and elevated (≥8.0 mg/L serum CRP) [28,31].

### 2.5. Covariates

Biological sex was characterized as male/female with male as the reference group. Age categories were defined as 18–34 years (reference), 35–64 years, and ≥65 years. The following race/ethnicity categories were used: non-Hispanic White (reference), non-Hispanic Black, Mexican-American/other Hispanic, and other races including multi-racial. Poverty status was categorized as income 200%+ poverty guidelines (reference), less than 200% poverty to 100% poverty, and under the poverty line (below 100% poverty). BMI was determined using anthropometric measures (height and weight) from the NHANES examination data and categorized as underweight (below 18.5), normal or healthy weight (18.5–24.9, reference), overweight (25.0–29.9), and obese (30.0 and above) [32].

### 2.6. Statistical Analyses

To describe the study sample, we generated frequency and proportion for each categorical variable. For bivariate analyses, we conducted second-order Rao and Scott tests to assess the independence between categorical variables and reported odds ratios (ORs) to quantify their associations. Potential covariates that presented statistically significant ORs with depression (*p* ≤ 0.05) were included in subsequent adjusted multivariate logistic regressions.

A series of multiple logistic regression analyses were conducted to estimate the association between depression and current e-cigarette use (non-smoking/never-vaping individual (0) vs. current e-cigarette user (1)). Firstly, we assessed the main effect of current e-cigarette use on depression (main effect model). Secondly, we assessed the simple slope of current e-cigarette use on depression using samples stratified by CRP category (reference vs. elevated; simple slope model). Lastly, to test the difference in simple slopes across CRP cut-points, we included e-cigarette use, CRP level, and their interaction in the logistic regression (full model). A significant interaction term indicated the odds of depression among current e-cigarette users vary by CRP level. We conducted similar analyses to estimate the association between depression and different e-cigarette use types (non-smoking/never-vaping individual (0) vs. exclusive e-cigarette user/former smoker e-cigarette user/dual user (1), respectively). To account for inflated Type I error, we controlled for false discovery rate (FDR) when claiming statistical significance (FDR *p* ≤ 0.05) [33]. Adjusted ORs along with 95% confidence intervals were reported. 

All multiple logistic regression analyses were adjusted for sex, poverty status, and BMI. We also reported results for unadjusted analyses for comparison. Sampling weights entered all analyses so that results are generalizable to the U.S. civilian noninstitutionalized resident population [34]. All data analyses were performed using Stata 16.0 (StataCorp LLC, College Station, TX, USA) and FDR corrected *p*-values were determined in RStudio version 1.4.1717 (RStudio PBC, Boston, MA, USA).

## 3. Results

As shown in Table 2, the unadjusted bivariate analyses demonstrate different relationships between independent variables of interest and current vaping status, depression score, and elevated serum CRP: (1) e-cigarette use (i.e., exclusive users, dual users, and former smokers who currently vape assessed as a single category) was associated with depression, sex, race, poverty status, and BMI; (2) depression (PHQ-9 score ≥ 10) was associated with e-cigarette use, elevated CRP, sex, poverty status, and BMI; and (3) elevated CRP (≥8 mg/L) was associated with depression, sex, race, poverty status, and BMI but not with e-cigarette use. E-cigarette users were significantly more likely to be depressed than the non-smoking/never-vaping reference population (16.7% and 5%, respectively, *p* < 0.001). Unadjusted analyses found an OR of 3.78 (95% Confidence Interval [CI]: 2.59, 5.52) for depression among all e-cigarette users. The OR for elevated CRP among current e-cigarette users was not significant in bivariate analyses (OR 0.93 [95% CI: 0.55, 1.57]), as was true for elevated CRP among exclusive e-cigarette users (OR 1.30 [95% CI: 0.56, 3.04]) (data not presented in Table 2). 12.41% of exclusive e-cigarette users had elevated CRP while 9.8% of non-users had elevated CRP (*p* > 0.05).

Table 3 presents adjusted multivariable analyses for depression among all current e-cigarette users by serum CRP (unstratified, <8 mg/L, ≥8 mg/L). When adjusting for confounders (i.e., those independent variables found to be significant in bivariate analyses at *p ≤* 0.05), e-cigarette users were more likely to be depressed (OR = 3.61, 95% CI: 2.43, 5.36) independent of serum CRP (main effect model: unstratified column). Stratification by CRP level in the model did impact the odds of depression among e-cigarette users compared to the reference group of equivalent cut-point (simple slope models): e-cigarette users with CRP < 8 mg/L had an OR of 3.37 (95% CI: 2.06, 5.51); these odds doubled to 6.70 (95% CI: 2.48, 18.11) when CRP ≥ 8 mg/L. When all participants who reported doctor diagnosis of previous myocardial infarction, stroke, or coronary heart disease were dropped from the analysis (*n* = 324 with one of more of these conditions), the ORs were essentially unchanged: adjusted OR for depression unstratified = 3.44 (95% CI: 2.20, 5.40), CRP < 8 mg/L = 3.21 (95% CI: 1.83, 5.63), and CRP ≥ 8 mg/L = 6.97 (95% CI: 2.69, 18.08) with all ORs significant at *p* ≤ 0.05 (results not reported in tabular form). 

Table 4 shows odds of depression in adjusted multivariable analyses of serum CRP across different categories of e-cigarette use (main effect model and simple slopes models). Similar to the results presented in Table 3, a pattern of increased odds of depression was observed among all categories of e-cigarette users with elevated CRP levels. Among each e-cigarette use group, dual users consistently had the highest odds of depression at each CRP strata (odds were evaluated by assessing each e-cigarette category against the same non-smoking/never-vaping reference group and CRP category): CRP < 8 mg/L had an adjusted OR of 4.31 (95% CI: 2.35, 7.89), while those with CRP ≥ 8 mg/L had an adjusted OR of 7.37 (95% CI: 1.85, 29.41). The pattern of increased odds of depression at the elevated CRP cut-point was also observed for depression among former smokers who are now exclusive e-cigarette users: OR = 2.78 (95% CI: 1.16, 6.69) and OR = 4.92 (95% CI: 0.46, 52.32), based on reference (<8 mg/L) and elevated (≥8 mg/L) CRP levels, respectively. When vaping individuals with any history of combustible tobacco use (i.e., current dual users and former smokers) were excluded from the population of e-cigarette users, analyses still identified the previously described pattern between depression, e-cigarette use, and inflammation persisted. After adjusting for confounders, exclusive e-cigarette users had an OR for depression of 2.40 (95% CI: 1.10, 5.26), independent of serum CRP (unstratified). Stratification by CRP level impacted odds of depression among exclusive e-cigarette users as follows: among e-cigarette users with CRP < 8 mg/L the OR = 1.91 (95% CI: 0.78, 4.69), whereas exclusive e-cigarette users with elevated CRP ≥ 8 mg/L were 5 times more likely to be depressed than the non-smoking/never-vaping reference group with elevated CRP (OR = 5.09, 95% CI: 1.44, 18.02; uncorrected *p*-value < 0.05, FDR adjusted *p*-value = 0.051).

Results for a full logistic regression model indicate that the association between depression and all current e-cigarette use status was not moderated by CRP level (*p*-value = 0.361). Similar findings were found when assessing the effect of distinct e-cigarette use type (exclusive e-cigarette use, dual user, and former smoking e-cigarette user) on depression. Results indicate these associations were not moderated by CRP (see Table 5, all FDR *p*-values > 0.05).

## 4. Discussion

It is well accepted that smokers are more likely to experience depression compared to non-smokers [35,36], and a number of studies have found that e-cigarette users also are at greater risk for depression [4,5,6,37]. Separately, both vaping and depression have been independently associated with increased inflammation [11,12,38]. Using data from a nationally representative sample of the U.S. population, we have found evidence consistent with early findings of current e-cigarette use being associated with increased likelihood of depression, and evidence of inflammation being associated with depression. Systemic inflammation, as defined as elevated CRP, was not found to be associated with current e-cigarette use. However, among all categories of e-cigarette use with elevated CRP, a pattern of increased odds of depression compared to the reference group of non-smoking/never-vaping individuals was observed, indicating the association between e-cigarette use and depression varies by stratified CRP level, albeit not significantly. This pattern persisted for odds of depression among exclusive e-cigarette users with elevated CRP, suggesting a relationship between vaping and depression irrespective of current or previous history of tobacco product use. To our knowledge, this is the first evidence that systemic inflammation may be involved in the relationship between depression and e-cigarette use as yet unexplained. Importantly, these associations remained when controlling for characteristics that are highly associated with depression such as sex, poverty status, and BMI [38,39,40,41]. However, it is important to note that inflammation was *not* found to moderate the association between vaping and depression in full model analyses.

Our findings from bivariate analyses are consistent with, and seemingly build upon, reports of independent associations between e-cigarette use and depression, and depression and elevated CRP [4,5,12,37,38], and a lack of evidence for a significant association between elevated CRP and e-cigarette use [42,43]. Similar to previous studies, we found that fewer women use e-cigarettes, yet are at elevated risk for depression (50%) and serum CRP ≥ 8.0 mg/L (100%) compared to men (Table 2) [38,44,45,46,47]. Among all current e-cigarette users, bivariate analyses did not identify an association between elevated CRP and vaping (OR = 0.93); adjusted analyses increased the odds of inflammation among this population, albeit not significantly (OR = 1.02, 95% CI: 0.59, 1.78). While also not powered for statistical significance, among exclusive e-cigarette users there was a greater likelihood of increased CRP, perhaps suggesting that e-cigarette use alone may contribute to systemic inflammation (OR = 1.30, 95% CI: 0.56, 3.04). Currently, the mechanisms influencing the relationship between vaping and inflammation are unknown. While we did not find evidence of an independent relationship between e-cigarette use and elevated CRP, the pattern observed in the simple slopes models leads us to postulate that these variables may still be linked in some way.

The main effect models demonstrate increased odds of depression for all categories of e-cigarette use, thus supporting a significant association between depression and vaping. Simple slopes models indicate the consistent pattern of increased ORs for depression at the elevated CRP cut-point compared to the reference cut-point. Although not statistically significant, the observed pattern presented in this paper is previously unreported and suggests a possible relationship between e-cigarette use, depression, and inflammation. These results concerning the association between current e-cigarette use and depression varying by stratified CRP level are similar to those found in a separate set of analyses using the 2015–2016 NHANES dataset that showed smoking and depression varied by stratified CRP level. These previous analyses also indicated individuals with elevated serum CRP who exclusively smoked cigarettes were over 3 times more likely to be depressed than nonsmokers with CRP ≥ 8.0 mg/L [24]. This similarity in the relationship between systemic inflammation, traditional cigarette and/or e-cigarette use, and depression may point to a potential shared biological mechanism facilitating these associations. Interestingly, dual users (individuals who routinely use both traditional cigarettes and e-cigarettes) were most likely to be depressed among all the categories of e-cigarette users with elevated CRP. However, when testing the difference in simple slopes across CRP cut-points with e-cigarette use, CRP level, and their interaction in the full model logistic regression included, inflammation does not moderate the relationship between vaping and depression.

Several limitations of this study should be noted, including the fact that NHANES is cross-sectional in nature. This limited the ability to draw causal inferences, or to determine the directionality of the association (i.e., whether depression leads to vaping or vaping leads to depression). Additionally, the small sample size, particularly among exclusive e-cigarette users, likely contributed to the wide confidence intervals associated with the determined odds ratios. The reliance on self-reported data for categorization of e-cigarette use, which increases the possibility of participant recall bias is another limitation. The use of a robust biomarker, such as cotinine would be preferred. However, cotinine only provides a snapshot of recent product use, and nicotine is widely present in both combustible cigarettes and e-cigarettes, which would preclude being able to classify which type of tobacco product was being used [48]. Additionally, the relatively short half-life of cotinine (<16 h) would hinder the ability to identify users who had not recently used either tobacco delivery system [49]. To confidently verify exclusive e-cigarette users, we would need to cross reference cotinine with a tobacco-specific analyte (like tobacco-specific nitrosamines) to rule out the consumption of combustible tobacco. By defining exclusive e-cigarette users as those who reported smoking less than 100 cigarettes in their lifetime, there is a possibility that members of the exclusive e-cigarette group had some minimal smoking experience. However, we utilized the CDC’s accepted definitions for smoker vs. nonsmoker based on the variables available in the NHANES dataset [50]. Moreover, the decision to exclude current and former smokers from the reference group results in a reference group that is an ideal control group for exclusive e-cigarette users. We do acknowledge that this choice may result in a less-than-perfect reference group for some subcategories of current e-cigarette users (particularly dual users and former smokers). To this end, we also ran analyses with a reference group that only excluded ever vaping individuals and found similar results (not shown). Ultimately, our decision to remove smokers was done to ensure that the reference group contained individuals with no history of active smoking or vaping. Furthermore, while CRP serves as a clinically validated marker of systemic inflammation, it is a biomarker that is associated with numerous diseases and pathologies that are accompanied by inflammation. Changes in serum CRP have been shown to be associated with age, health status, medication, diet, and other factors that were not accounted for in the described models [51,52,53,54]. Additional analyses, however, indicate how the ORs for depression are influenced by the exclusion of participants who reported doctor diagnosis of previous myocardial infarction, stroke, or coronary heart disease as participants with these conditions have been excluded in similar analyses in the literature [42]. Depression has also been found to be a common comorbidity of chronic diseases such as these [55]. Even with these participants removed, the pattern indicating increased likelihood of depression among current e-cigarette users with elevated systemic inflammation persists. Finally, we acknowledge that there are other potential confounding factors (e.g., education, marital status, alcohol use, physical activity, and social support) that have not been thoroughly explored in this study. We suggest that future analyses with a larger sample size take these possible covariates into account. 

In addition to addressing these limitations, future investigations using additional inflammatory markers are needed to elucidate whether specific aspects of e-cigarette liquids and/or aerosols are associated with inflammation. If e-cigarette use is found to lead to increased risk of depression via systemic inflammation, targeting the most inflammatory components of inhaled e-cigarette aerosols for regulation and bans could allow for improved mental health outcomes among e-cigarette users. Alternatively, if depression enhances the likelihood of e-cigarette use, this may have implications for policies and practices targeting individuals suffering from depression.

## 5. Conclusions

Despite these limitations, the present findings add to the growing literature on depression, and more specifically to its possible link to different personal variables, tobacco-use behaviors, and elevated systemic inflammation [38,42,52,56,57]. The results presented do not provide further clarification for the relationship between e-cigarette use and systemic inflammation, but the pattern observed suggests that inflammation may be associated with, predict, or contribute to depression among e-cigarette users as yet unknown. The suspected relationship between vaping, adverse mental health outcomes, and systemic inflammation needs to be investigated further, preferably with a larger sample size. With the remarkably rapid uptake of e-cigarette usage around the world as an alternative tobacco product, it is imperative to identify the health consequences of its use as well as the mechanisms of adverse health effects [1]. As the literature on the potential negative impacts of vaping continues to expand, future studies should continue to explore the link between e-cigarette use and mental health risk. As previously noted, our findings provide evidence that make us question the nature of the role that systemic inflammation may play in the association between depression and e-cigarette use, but the cross-sectional nature of these data highlights the need for both longitudinal studies and mechanistic research to understand the biologic underpinnings of this association. The pattern of increased depression risk among e-cigarette users with elevated systemic inflammation persists even in the absence of or with minimal combustible tobacco product exposure, which suggests the association observed among all current e-cigarette users is likely not driven entirely by a history of, or concurrent smoking. Should inflammation be found to play a key role in the association between e-cigarette use and depression in future studies, it may have substantial implications both for mental health interventions and for the regulation of e-cigarettes worldwide.

## Figures and Tables

**Table 1:** Characteristics of the NHANES 2015–2016 & 2017–2018 Study Population (*n* = 4961).

Characteristic	*n*	Weighted %
Age		
18–34 years	1536	31.39
35–64 years	2461	51.92
≥65 years	964	16.69
Sex		
Male	2043	42.98
Female	2918	57.02
Race		
Non-Hispanic White	1595	63.77
Non-Hispanic Black	1011	10.49
Mexican American/Other Hispanic	1402	15.78
Other Races, including Multi-Racial	953	9.96
Poverty Status		
Below 100% poverty	959	12.07
Less than 200% poverty to 100% poverty	1261	18.52
200%+ poverty guidelines	2741	69.4
Body Mass Index		
Underweight (<18.5)	77	1.73
Normal weight (18.5–24.9)	1338	27.58
Overweight (25.0–29.9)	1554	30.71
Obese (30+)	1992	39.98
Current E-Cigarette Use		
Yes	443	10.15
No	4518	89.85
Current E-Cigarette Use Categories		
Exclusive E-Cigarette Users	110	1.92
Former Smoker E-Cigarette Users	92	2.6
Dual Users	241	5.63
Non-Smoking, Never-Vaping Persons	4518	89.85

**Table 2:** Bivariate Analyses Between Rates of Depression, Current E-cigarette Status, and Elevated CRP and Independent Variables.

Variable	Weighted % Depressed; Unadjusted Depression (OR, 95% CI)	Weighted % of Current E-cigarette Users; Unadjusted Current E-Cigarette Users (OR, 95% CI)	Weighted % with CRP ≥ 8.0 mg/L; Unadjusted CRP ≥ 8.0 mg/L (OR, 95% CI)
Depression PHQ Score			
<10		9.0%; Ref	9.4%; Ref
≥10		27.3%; **3.78 (2.59, 5.52)**	15.3%; **1.75 (1.24, 2.46)**
Current E-cigarette User			
No	5.0%; Ref		9.8%; Ref
Yes	16.7%; **3.78 (2.59, 5.52)**		9.2%; 0.93 (0.55, 1.57)
CRP Level			
<8.0 mg/L serum CRP	5.8%; Ref	10.2%; Ref	
≥8.0 mg/L serum CRP	9.8%; **1.75 (1.24, 2.46)**	9.6%; 0.93 (0.55, 1.57)	
Sex			
Male	5.0%; Ref	14.3%; Ref	6.6%; Ref
Female	7.1%; **1.46 (1.05, 2.02)**	7.0%; **0.45 (0.35, 0.60)**	12.1%; **1.96 (1.53, 2.50)**
Race			
NH White	5.9%; Ref	10.8%; Ref	8.9%; Ref
NH Black	6.8%; 1.16 (0.84, 1.59)	9.9%; 0.91 (0.66, 1.24)	14.2%; **1.69 (1.30, 2.21)**
Hispanic	6.5%; 1.11 (0.69, 1.79)	7.3%; **0.64 (0.49, 0.85)**	11.7%; **1.35 (1.12, 1.64)**
Other	7.2%; 1.23 (0.67, 2.26)	10.6%; 0.97 (0.62, 1.54)	7.2%; 0.79 (0.53, 1.17)
Poverty Status			
200%+ poverty guidelines	4.4%; Ref	8.1%; Ref	8.4%; Ref
Less than 200% poverty to 100% poverty	8.8%; **2.11 (1.50, 2.98)**	13.1%; **1.71 (1.13, 2.58)**	12.1%; **1.50 (1.12, 2.01)**
Below 100% poverty	12.8%; **3.22 (2.45, 4.24)**	17.2%; **2.35 (1.64, 3.37)**	13.7%; **1.73 (1.30, 2.30)**
BMI			
Normal weight (18.5–24.9)	5.5%; Ref	10.9; Ref	2.9%; Ref
Underweight (<18.5)	12.2%; 2.40 (0.49, 11.78)	22.9%; **2.43 (1.27, 4.65)**	6.5%; 2.32 (0.47, 11.47)
Overweight (25.0–29.9)	4.9%; 0.88 (0.63, 1.25)	8.9%; 0.80 (0.58, 1.11)	4.9%; 1.74 (0.95, 3.20)
Obese (30+)	7.5%; **1.41 (1.00, 1.97)**	10.0%; 0.91 (0.68, 1.22)	18.3%; **7.52 (4.22, 13.39)**

Note: Odds ratios in bold are statistically significant (*p* ≤ 0.05).

**Table 3:** Odds of Depression in Multivariable Analysis Among All Current E-cigarette Users ^a^ Stratified by Level of CRP.

Variable	Main Effect Model	Simple Slopes Models	
	Unstratified CRP (OR, 95% CI) ^b^	CRP < 8.0 mg/L (OR, 95% CI) ^b^	CRP ≥ 8.0 mg/L (OR, 95% CI) ^b^
Current E-Cigarette User			
No	Ref	Ref	Ref
Yes	**3.61 (2.43, 5.36)**	**3.37 (2.06, 5.51)**	**6.70 (2.48, 18.11)**
Sex			
Male	Ref	Ref	Ref
Female	**1.59 (1.16, 2.18)**	**1.45 (1.08, 1.93)**	2.77 (0.59, 12.99)
Poverty Status			
200%+ poverty guidelines	Ref	Ref	Ref
Less than 200% poverty to 100% poverty	**1.86 (1.30, 2.66)**	**2.01 (1.38, 2.93)**	1.04 (0.30, 3.61)
Below 100% poverty	**2.69 (2.03, 3.56)**	**2.48 (1.82, 3.38)**	**3.16 (1.09, 9.10)**
BMI			
Normal weight (18.5–24.9)	Ref	Ref	Ref
Underweight (<18.5)	1.78 (0.27, 11.76)	1.88 (0.25, 14.20)	4.07 (0.10, 168.40)
Overweight (25.0–29.9)	0.97 (0.66, 1.43)	0.94 (0.63, 1.40)	6.49 (0.69, 60.64)
Obese (30+)	1.41 (0.97, 2.07)	1.20 (0.81, 1.79)	**17.84 (2.18, 146.35)**

Note: Odds ratios in bold are statistically significant (FDR *p* ≤ 0.05). ^a^ ‘All Current E-Cigarette Users’ consists of all individuals who reported using an e-cigarette in the last 30 days including exclusive e-cigarette users, dual users, and former smokers who currently vape exclusively. ^b^ ORs have been adjusted for sex, poverty status, and BMI.

**Table 4:** Odds of Depression in Multivariable Analysis Among Different Categories of E-cigarette Users and Levels of CRP.

Variable	Main Effect Model	Simple Slopes Models	
	Whole Sample (OR, 95% CI)	CRP < 8.0 mg/L (OR, 95% CI)	CRP ≥ 8.0 mg/L (OR, 95% CI)
All Current E-Cigarette Users ^a^			
Unadjusted	**3.78 (2.59, 5.52)**	**3.54 (2.24, 5.61)**	**6.06 (2.41, 15.21)**
Adjusted	**3.61 (2.43, 5.36)**	**3.37 (2.06, 5.51)**	**6.70 (2.48, 18.11)**
Exclusive E-Cigarette Users			
Unadjusted	**2.69 (1.23, 5.89)**	2.14 (0.87, 5.23)	**6.00 (1.15, 31.27)**
Adjusted	**2.40 (1.10, 5.26)**	1.91 (0.78, 4.69)	5.09 (1.44, 18.02) ^b^
Dual Users			
Unadjusted	**4.76 (2.99, 7.57)**	**4.56 (2.65, 7.84)**	**6.64 (2.49, 17.68)**
Adjusted	**4.50 (2.70, 7.48)**	**4.31 (2.35, 7.89)**	**7.37 (1.85, 29.41) ^c^**
Former Smokers Using E-Cigarettes Exclusively			
Unadjusted	**2.67 (1.40, 5.07)**	**2.58 (1.12, 5.91)**	4.47 (0.53, 37.79)
Adjusted	**2.78 (1.38, 5.59)**	**2.78 (1.16, 6.69)**	4.92 (0.46, 52.32) ^d^

Note: Odds ratios in bold are statistically significant (FDR *p* ≤ 0.05). The OR for each variable is for a weighted logistic regression model in which the association between depression and the concerned variable and covariates (i.e., sex, poverty status, and BMI) were examined. The reference group is made up of non-smoking/never-vaping individuals. ^a^ The group ‘All Current E-Cigarette Users’ consists of all individuals who reported using an e-cigarette in the last 30 days including participants in the groups subsequently analyzed independently (exclusive e-cigarette users, dual users, and former smokers who currently vape exclusively). ^b^ 37 observations were dropped from the BMI ‘Normal Weight’ category because the outcome was predicted perfectly and the BMI ‘Obese’ category was omitted due to collinearity. ^c^ 1 observation was not used from the BMI ‘Underweight’ category because the outcome was predicted perfectly. ^d^ 36 observations were dropped from the BMI ‘Normal Weight’ category because the outcome was predicted perfectly and the BMI ‘Obese’ category was omitted due to collinearity.

**Table 5:** Unadjusted and Adjusted Odds Ratios and Their Confidence Intervals (CI) from Logistic Regression Analyses Estimating the Interaction Effect of Current E-cigarette Use and C-reactive Protein on Depression.

Interaction	Full Model
	Whole Sample (OR, 95% CI), *p*-Value
CRP X Current E-Cigarette Use ^a^	
Unadjusted	1.71 (0.52, 5.57), 0.361
Adjusted	1.45 (0.41, 5.05), 0.552
CRP X Exclusive E-Cigarette Use	
Unadjusted	2.80 (0.43, 18.31), 0.670
Adjusted	2.43 (0.38, 15.47), 0.998
CRP X Dual User	
Unadjusted	1.46 (0.43, 4.98), 0.670
Adjusted	1.29 (0.34, 4.88), 0.998
CRP X Former Smoker Using E-Cigarettes Exclusively	
Unadjusted	1.73 (0.13, 23.64), 0.670
Adjusted	1.00 (0.72, 2.03), 0.998

Note: No odds ratios are statistically significant (all FDR *p*-values > 0.05). The OR for each interaction variable is for a weighted logistic regression model in which the association between depression, each concerned variable and their interaction, and covariates (i.e., sex, poverty status, and BMI) were examined. ^a^ The group ‘All Current E-Cigarette Users’ consists of all individuals who reported using an e-cigarette in the last 30 days including participants in the groups subsequently analyzed independently (exclusive e-cigarette users, dual users, and former smokers who currently vape exclusively).

## Data Availability

The data presented in this study are openly available from the National Center for Health Statistics—National Health and Nutrition Examination Survey online database at https://wwwn.cdc.gov/nchs/nhanes/.
